# Supplementary material for: Plant–Microbes Interaction: Exploring the Impact of Cold-Tolerant Bacillus Strains RJGP41 and GBAC46 Volatiles on Tomato Growth Promotion through Different Mechanisms
Source: Biology (Basel). 2023 Jun 30;12(7):940. doi: 10.3390/biology12070940 (PMC10376619; doi:10.3390/biology12070940)
Supplement: Supplementary file 1 [file biology-12-00940-s001.zip › biology-2435711-supplementary.pdf]

Table S1: Primers used in this study.

No	Gene	Gene	Primer sequence
1	Cytokinin oxidase/dehydrogenase	*CKX2*-F<br>*CKX2*-R	TCCCTTATGTTGATGTCTCAGC<br>TTACTGATTTGAGGGCCGTG
2	Auxin-responsive protein	*IAA4*-F<br>*IAA4* -R	AGGCAACAGAGCTTAGATTGG<br>GGCAACAGGTGGAGTTTTG
3	Auxin response factor 10A	*ARF10A*-F<br>*ARF10A*-R	TTCTCCATGTTCTGCAGGTG<br>TGGTAACTGAAACTGGCTGTC
4	Gibberellin 2-oxidase	GA_2Ox_2-F<br>GA_2Ox_2-R	TGTTGGCGGAGGGATTAAAG<br>AGCATGGCGGATAGTGATTC
5	Ethylene response factor A	*ERFA2*-F<br>*ERFA2*-R	CGGTTAGAGTGACGGTTAAGAG<br>TCATAACATTTGGTCCCCGG
6	Expansion	*EXP1*-F<br>*EXP1*-R	CACATGCTACATTTTACGGCG<br>GTTCAAAACAGGCTCCACAAC
